# Characterization of CAR-T cell factors that contribute to myeloid cell activation

**DOI:** 10.1038/s41434-026-00620-6

**Published:** 2026-05-26

**Authors:** Supreet Khanal, Md Kamal Hossain, Joseph Fischer, Santosh Panthi, Alan Baer, Nirjal Bhattarai

**Affiliations:** 1 Tumor Vaccine and Biotechnology Branch, Office of Cellular Therapy and Human Tissues, Office of Therapeutic Products, Center for Biologics Evaluation and Research, U.S. FDA, Silver Spring, MA USA; 2 Present Address: Division of Biotechnology Review and Research III, Office of Biotechnology Product, Center for Drug Evaluation and Research, U.S. FDA, Silver Spring, MD USA; 3 Present Address: Cell Therapy Research and Development, AstraZeneca, Gaithersburg, MD USA; 4 Present Address: Therapeutic Development Branch, Division of Preclinical Innovation, National Center for Advancing Translational Sciences (NCATS), NIH, Rockville, MD USA

**Keywords:** Immunotherapy, Adaptive immunity

## Abstract

Chimeric antigen receptor (CAR) T cell therapies have shown remarkable success in the treatment of hematologic cancers; however, their use is often accompanied by inflammatory toxicities, including cytokine release syndrome (CRS) and immune effector cell-associated neurotoxicity syndrome (ICANS). These toxicities, ranging from mild to life-threatening, are partly driven by bystander myeloid cell activation (BMCA) and the subsequent release of pro-inflammatory cytokines such as IL-6 and IL-1β. Although previous studies have described the individual contributions of GM-CSF, IFN-γ, and TNFα secreted by CAR-T cells, a comprehensive characterization of CAR-T-derived inflammatory factors has been lacking. In this study, we characterized the soluble factors secreted by activated CAR-T cells derived from human peripheral blood and assessed their role in BMCA. Comparative cytokine analyses across human T cell subsets, including CAR-T cells, identified multiple candidates involved in BMCA. Antibody-mediated neutralization confirmed that four factors, GM-CSF, IFN-γ, TNFα, and GPIbα, play dominant roles in driving BMCA. Furthermore, siRNA-mediated knockdown of these factors in CAR-T cells significantly reduced BMCA without impairing their anti-tumor activity. These findings are consistent with prior reports on the inflammatory roles of GM-CSF, IFN-γ, and TNFα during CAR-T cell therapy and, importantly, identify GPIbα as a previously unrecognized contributor to CAR-T-associated inflammatory toxicities. Targeting these factors through antibody blockade or genetic modification may represent a promising strategy to mitigate inflammatory toxicities and improve the safety of CAR-T cell therapies.

## Introduction

Chimeric antigen receptor (CAR) T cell therapies have revolutionized the treatment of hematologic malignancies, offering high rates of remission for otherwise refractory cancers. However, these therapies are frequently associated with life-threatening inflammatory toxicities, most notably cytokine release syndrome (CRS) and immune effector cell-associated neurotoxicity syndrome (ICANS), collectively referred to as CAR-T-related inflammatory toxicities [1, 2]. CRS arises from the hyperactivation and proliferation of CAR-T cells, which trigger a cascade of immune responses involving the release of pro-inflammatory cytokines such as interleukin-6 (IL-6) and interleukin-1β (IL-1β) from various immune cells [3]. This cytokine surge can result in systemic inflammation, manifesting as flu-like symptoms, high fever, and in severe cases, multi-organ dysfunction. Neurotoxicity, a distinct but overlapping toxicity, involves disruptions to the central nervous system (CNS) and can lead to confusion, seizures, cerebral edema, or death [1, 2, 4]. The over-production of inflammatory cytokines associated with CRS can also lead to other toxicities [5–7]. The underlying mechanisms for development of these inflammatory toxicities are not well understood, and the severity varies considerably between patients ranging from low-grade fever to severe inflammation resulting in death [8].

Current strategies for managing CAR-T cell therapy-associated inflammatory toxicities primarily rely on immunomodulatory drugs such as tocilizumab, anakinra and corticosteroids [8–10]. Tocilizumab, an IL-6 receptor antagonist, is effective against cytokine release syndrome (CRS) [11], but has limited impact on neurotoxicity [12, 13] partly due to its inability to effectively cross the blood-brain barrier and inhibit IL-6 activity in the central nervous system [14]. IL-1β, which is upregulated in severe CRS, disrupts the blood-brain barrier [7, 15], and precedes IL-6 expression in mice [16], making it a promising target for mitigating CAR-T-related toxicities. The FDA-approved IL-1 receptor antagonist, anakinra, has been used to inhibit IL-1β activity in patients [5, 17, 18], and prevented both CRS and neurotoxicity in mice [15, 16]. Inhibition of IFNγ and TNFα has been also shown to be effective in some cases when patients do not respond to other treatments [19, 20]. While these immunomodulatory strategies are standard treatments; they are expensive, show variable patient responses, and can introduce additional toxicities. Furthermore, these agents primarily target inflammatory cytokines such as IL-6 and IL-1β, which are primarily produced by activated myeloid cells (e.g., macrophages, monocytes) rather than CAR-T cells [16]. The mechanisms contributing to bystander myeloid cell activation leading to inflammatory toxicities during CAR-T cell therapy remain poorly understood.

Activation of CAR-T cells leads to the secretion of a variety of soluble factors, including cytokines and chemokines. While some of these factors are essential for CAR-T cell expansion, persistence, and antitumor activity, others can drive inflammatory toxicities such as CRS and ICANS [4]. Previous studies have demonstrated that IFNγ [21, 22], GM-CSF [23, 24], and TNFα [20, 25, 26] produced by activated CAR-T cells play key roles in the activation of bystander myeloid cells and the amplification of inflammatory responses. Neutralization or genetic ablation of pro-inflammatory cytokines such as IFNγ, GM-CSF and TNFα in preclinical models has been shown to significantly reduce CAR-T cell-associated toxicities. However, complete deletion of these cytokines may not always be optimal. For instance, IFNγ plays a critical role in enhancing CAR-T cell cytotoxicity, modulating the tumor microenvironment, and recruiting additional immune cells [22, 27]. Its absence could potentially compromise anti-tumor efficacy, particularly in solid tumors or immunosuppressive settings where IFNγ-mediated signaling supports therapeutic activity as studies have demonstrated that intact IFNγ signaling is required for CAR-T cell efficacy against solid tumors [22, 27].

Moreover, targeting a single cytokine may not be sufficient to fully mitigate inflammatory toxicities. For example, GM-CSF neutralization in mice did not consistently suppress IL-6 and IL-1β secretion from myeloid cells [23, 28]. While macrophages from GM-CSF knockout (KO) mice showed reduced expression of both IL-6 and IL-1β [29], studies in human cells revealed that GM-CSF neutralization lowered IL-6 levels [24] but had limited effect on IL-1β production [30]. These observations suggest that a more comprehensive or combinatorial approach may be necessary to effectively modulate the complex cytokine networks involved in CAR-T cell associated toxicities. Identifying the inflammatory mediators involved in CAR-T cell therapy is essential for understanding the mechanisms underlying treatment-associated toxicities and for developing targeted strategies to mitigate them.

In this study, we performed a comprehensive assessment of the drivers of bystander myeloid cell activation during CAR-T cell therapy and identified major CAR-T cell-derived factors that contribute to inflammatory toxicity.

## Materials and methods

### Cell lines

THP-1 and Daudi cells were obtained from ATCC, and HEK293T were obtained from Cell Genesys. THP-1 cells were cultured in IMDM medium (ATCC) supplemented with 100 IU/mL penicillin, 10 µg/mL streptomycin, and 20% heat-inactivated fetal bovine serum (HI-FBS; Atlanta Biologicals). Daudi cells were cultured in RPMI 1640 medium supplemented with 100 IU/mL penicillin, 10 µg/mL streptomycin, 2 mM GlutaMax (Thermo), and 10% HI-FBS. HEK293T cells were cultured in DMEM medium (Lonza) supplemented with 100 IU/mL penicillin, 10 µg/mL streptomycin, 2 mM GlutaMax (Thermo), and 10% HI-FBS. Nalm6 (RRID:CVCL_0092) cells expressing GFP/luciferase with beta-2 microglobulin (B2M) knockout were described previously [31]. All cell lines were maintained at 37⁰C with 5% CO_2_.

### Primary cells

Buffy coats from de-identified healthy donors were obtained from the National Institutes of Health (NIH) Blood Bank. All donors provided written consent for the use of their samples in laboratory research, and this study was exempted by the FDA IRB, Research Involving Human Subjects Committee (RIHSC). No subject selection criteria were used for healthy donors. Peripheral blood mononuclear cells (PBMCs) were isolated from fresh buffy coat using Ficoll gradient centrifugation (GE Healthcare). The isolated PBMCs were washed twice with 1X PBS and incubated overnight at 37 °C in complete RPMI 1640 medium supplemented with 2 mM L-glutamine, 100 IU/mL penicillin, 10 µg/mL streptomycin, and 10% HI-FBS in a humidified incubator with 5% CO₂. Suspension cells were used for T-cell isolation using a human T-cell isolation kit (Miltenyi Biotech). Adherent monocytes were washed three times with 1X PBS and treated with trypsin for 3 min at 37 °C. Trypsin was neutralized using complete RPMI medium. Cell purity was assessed by flow cytometry using antibodies against T cells (CD3-PE; BioLegend), B cells (CD19-APC; BD Biosciences), and monocytes (CD11b-V450; BD Biosciences). Cell populations with >90% purity were routinely obtained. Isolated primary T cells were cultured in complete RPMI, and primary monocytes were cultured in complete IMDM.

### Anti-CD19 CAR expressing lentiviral vector

Lentiviral vector used in this study was manufactured as previously described [31, 32]. The concentrated vector was aliquoted and stored at –80 °C. Viral titers were determined using Jurkat cells. A total of 1 × 10⁶ Jurkat cells were transduced with serially diluted lentiviral vector in the presence of polybrene (AmericanBio) for 72 h. CAR expression on live cells (DAPI exclusion) was analyzed by flow cytometry (BD LSR Fortessa) using FITC-conjugated Protein L (AcroBio). Viral titer (T) was calculated using the formula: T = (P × N) / (D × V),

where T = titer (TU/mL), P = percentage of FITC-positive cells determined by flow cytometry, N = number of cells at the time of transduction, D = dilution factor, and V = total volume of viral inoculum.

### CAR-T cells

T cells isolated from PBMCs were activated using anti-CD3/CD28 activation reagent (StemCell Technologies) for 48–72 h in ImmunoCult-XF T-cell expansion medium (StemCell Technologies) supplemented with IL-7 (1 ng/mL; PeproTech), IL-2 (50 ng/mL; PeproTech), and 5% human serum (Sigma). Activated T cells were transduced with the anti-CD19 CAR lentiviral vector in the presence of polybrene at a multiplicity of infection (MOI) of 10. Spinoculation was performed at 1800 rpm for 1 h at 25 °C in 6-well non-tissue culture-treated plates. Following spinoculation, cells were incubated at 37 °C for 6 h, after which fresh medium was added, and the cells were expanded at 1 × 10⁶ – 2 × 10⁶ cells/mL for 14 days. Untransduced T cells underwent mock spinoculation without vector. CAR-T cells were assessed for viability, CD19-CAR expression (Protein L staining), and endotoxin levels prior to use.

### Cell count and viability

Cell count and viability were determined using automated cell counter (Countess II, ThermoFisher).

### Flow cytometry

Cells were incubated with Protein L for 1 h and washed twice with PBS. Data were acquired on a BD LSR Fortessa using single-stained controls for compensation. At least 10,000 total events were collected, and FlowJo software (Tree Star Inc.) was used for data analysis.

### Activation of T and CAR-T cells

Primary T and CAR-T cells were activated using anti-CD3/CD28/CD2 activation reagent (StemCell Technologies), phorbol 12-myristate 13-acetate (PMA; 50 ng/mL; Sigma) plus ionomycin (1 µg/mL; Sigma), or co-cultured with CD19⁺ target cells (Daudi or Nalm6) for 24 h at 37 °C. Supernatants were collected and centrifuged at 2000 × g for 10 min to obtain cell-free fractions.

### Myeloid cell activation assay

Primary human monocytes or THP-1 cells were used to assess myeloid cell activation. Supernatants from either unstimulated (US) or activated primary T and CAR-T cells were added to myeloid cells (1 × 10⁶ cells/mL) in a 96 well culture plate and incubated for 24 h. Myeloid cell activation (MCA) was assessed by quantifying IL-6 and IL-1β levels in the supernatant using ELISA. Percentage reduction in MCA was calculated by normalizing and comparing IL-6 and IL-1β levels to their respective controls.

### ELISA

IFNγ, GM-CSF, TNFα, IL-6 and IL-1β were quantified using ELISA kits (BD Biosciences). ID5 (Molecular Devices) or Varioskan Lux (Thermo Scientific) plate readers were used to measure absorbance.

### Cytokine array

Inflammatory cytokines in cell-free supernatants were profiled by Eve Technologies, Canada, using the Human Cytokine/Chemokine Assay.

### Cytotoxicity assay

Cytotoxicity was assessed as previously described [31, 32]. Briefly, CD19⁺ Nalm6 cells (target cells) expressing firefly luciferase were co-cultured with CAR-T cells (effector cells) at various effector-to-target (E:T) ratios in 96-well plates. As a negative control, Nalm6 cells were plated alone; as a positive control, Nalm6 cells were treated with 10% Triton X-100. After 24 h, an equal volume of Bright-Glo luciferase reagent (100 μL; Promega) was added, and luminescence was measured using a Varioskan plate reader (Thermo Scientific) in a white 96-well plate. Cytotoxicity was calculated using the formula: Cytotoxicity (% lysis) =100− [(Luminescence for co-culture sample/Luminesce for Nalm6 alone) ×100].

### Neutralization assay

Cell-free supernatants obtained from activated T or CAR-T cells were used for neutralization studies. Antibodies against IFNγ, GM-CSF, GPIbα, TNFα, IL-13, IL-16, IL-31, MIP-1α, MIP-1β, IP-10, I-309, M-CSF, IL-17A, and IL-17F were purchased from R&D Systems. A non-specific IgG antibody control was purchased from Santa Cruz Biotechnology. Optimal antibody concentrations for neutralization were determined using activated CAR-T supernatants; 25 µg/mL achieved >80% neutralization. For neutralization experiments, antibodies were added to activated supernatants and incubated for 2 h at room temperature. For combination neutralization assays, antibody concentrations were equally divided, ensuring the total did not exceed 50 µg/mL. Equal amounts of IgG control were included for each condition. Following neutralization, myeloid activation was assessed as described above.

### Recombinant GPIbα protein

The inflammatory potential of recombinant GPIbα protein was assessed using THP-1 cells. Recombinant GPIbα (25 µg/mL; R&D Systems) was incubated with resting THP-1 cells for 24 h. Activation was evaluated by measuring secreted IL-6 levels using ELISA.

### siRNA and gene expression analysis

Non-specific control siRNA and gene-specific siRNAs (*IFNG, GMCSF, GP1BA*, and *TNF*) were purchased from Thermo Fisher. Validated qPCR primers for the same genes were purchased from Origene. CAR-T cells were transfected with control or pooled gene-specific siRNAs using the Amaxa nucleofector system with the Human T Cell Nucleofector Kit (Lonza) according to the manufacturer’s protocol. The pooled siRNA strategy was adopted to model a multi-target mitigation approach, which can be easily implemented during CAR-T cell manufacturing workflow. mRNA and protein expression were analyzed 48 h post-transfection. For mRNA analysis, total RNA was extracted using the RNeasy Mini Kit (Qiagen) with DNase treatment (RNase-Free DNase Set, Qiagen). Complementary DNA (cDNA) was synthesized using the iScript cDNA Kit (Bio-Rad), and relative gene expression was determined and normalized to beta-2 microglobulin using the CFX96 Real-Time PCR Detection System (Bio-Rad). For protein analysis, siRNA-transfected cells were activated for 24 h, and supernatants were assessed by ELISA.

### Immunoblot

Samples were mixed with Laemmli sample buffer, heated at 95 °C for 5 min, separated on NuPAGE Bis-Tris gels by electrophoresis, and transferred to nitrocellulose membranes using the iBlot transfer system (Thermo Scientific). Membranes were blocked with 3% nonfat dry milk for 1 h at room temperature, followed by overnight incubation with anti-GPIbα antibody (R&D Systems). GPIbα was detected using SuperSignal West Dura (Thermo Scientific) and visualized on a Bio-Rad ChemiDoc MP imaging system. Images were quantified using ImageJ (NIH).

### Statistics

Two-sided Student’s t-tests were used to compare two groups, and one-way ANOVA was used for multiple groups. *P* values < 0.05 were considered statistically significant. GraphPad Prism was used for statistical analyses.

## Results

### Soluble factors secreted by activated T cells and CAR-T cells drive bystander myeloid cell activation

**Fig. 1:**
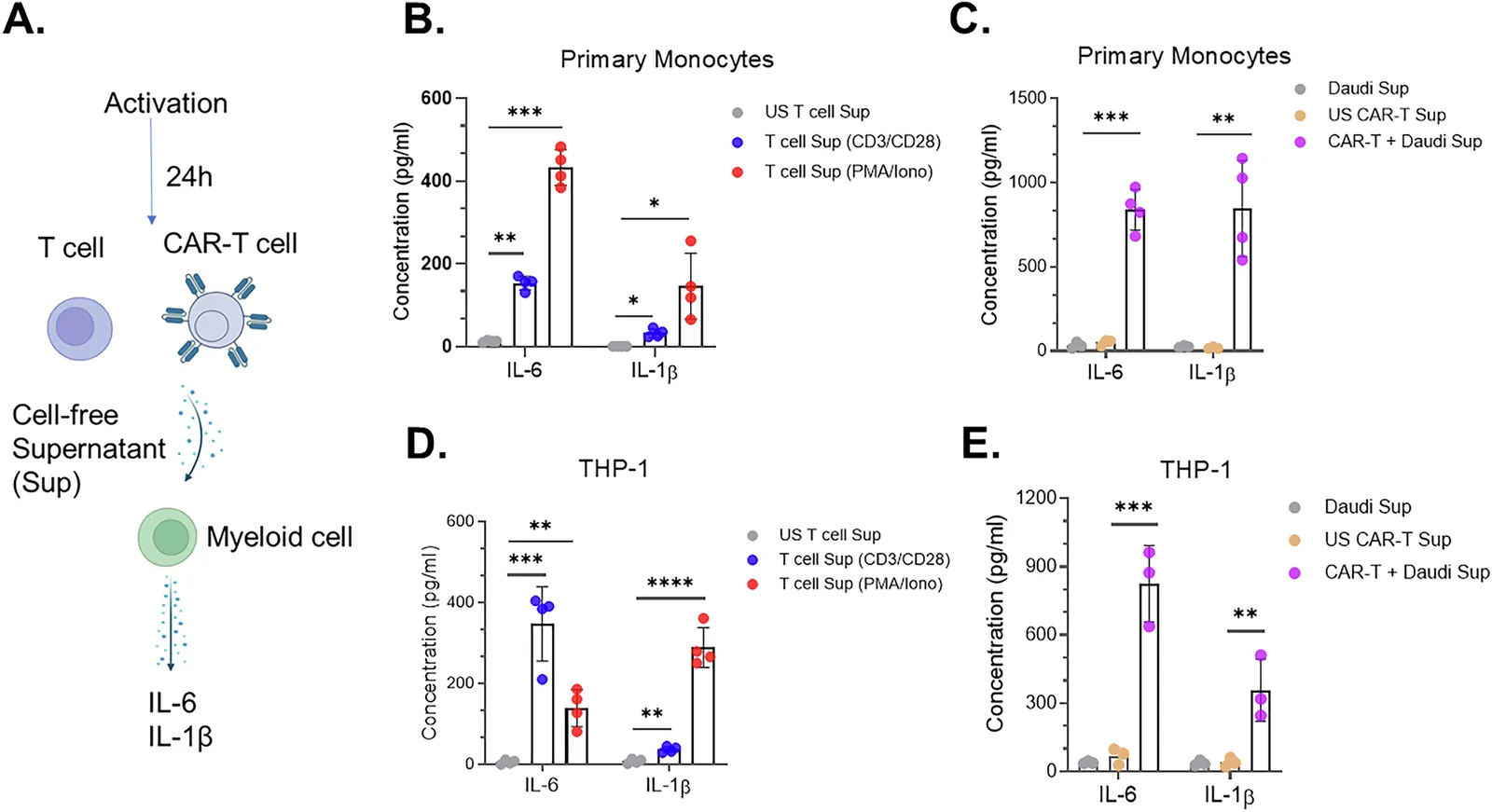
Soluble factors secreted by activated T cells and CAR-T cells drive bystander myeloid cell activation. **A** Schematic of experimental steps followed for myeloid cell activation assay. Primary human T or anti-CD19 CAR-T cells were activated and following 24 h of activation, cell-free supernatant was obtained and incubated with myeloid cells. Myeloid cell activation was measured after 24 h by assessing IL-6 and IL-1β secreted by myeloid cells in the supernatant. IL-6 and IL-1β secreted by primary monocytes following treatment with cell-free supernatants obtained from either unstimulated (US) or activated **B** T cells and **C** anti-CD19 CAR-T cells. IL-6 and IL-1β secreted by THP-1 monocytic cell line following treatment with cell-free supernatants obtained from either unstimulated (US) or activated **D** T cells and **E** anti-CD19 CAR-T cells. *P < 0.05, **P < 0.01, ***P < 0.001. Each dot represents one donor.

In vitro, chimeric antigen receptor (CAR) T cells can be activated through various methods, including T cell receptor (TCR)-dependent activation with anti-CD3/CD28 antibodies, TCR-independent activation with phorbol myristate acetate (PMA) and ionomycin (Iono), or CAR-mediated activation by co-culturing CAR-T cells with target cells. To determine whether soluble factors secreted by T cells upon activation via TCR-dependent, TCR-independent, or CAR-dependent mechanisms can induce bystander myeloid cell activation (BMCA), primary human T cells or anti-CD19 CAR-T cells were activated using anti-CD3/CD28, PMA/Iono, or co-culture with CD19⁺ Daudi cells. After 24 h, cell-free supernatants were collected and added to myeloid cells, either primary human monocytes or the THP-1 monocytic cell line. BMCA was then assessed after an additional 24 h by measuring IL-6 and IL-1β secretion from the myeloid cells (Fig. 1A).

Supernatants obtained from primary T cells activated with anti-CD3/CD28 or PMA/Iono contained high levels of IFN-γ, and GM-CSF compared with supernatants from unstimulated (US) T cells (Fig. S1A). Similarly, supernatants from anti-CD19 CAR-T cells co-cultured with Daudi cells contained high levels of IFN-γ and GM-CSF compared with supernatants from Daudi cells alone or US CAR-T cells (Fig. S1B). These findings confirm robust activation of T cells and CAR-T cells and the presence of IFN-γ and GM-CSF in the collected supernatants.

Next, primary human monocytes were treated with cell-free supernatants obtained from T and CAR-T cells. Supernatants from T cells activated via either TCR-dependent or TCR-independent methods, as well as from activated CAR-T cells, significantly induced IL-6 and IL-1β production in primary monocytes (Fig. 1B). In contrast, supernatants from US T cells, US CAR-T cells, or Daudi cells did not induce cytokine production (Fig. 1C), indicating that myeloid activation is specifically driven by factors secreted by activated, but not resting, T cells and CAR-T cells. Consistent with the data obtained from primary human monocytes, the THP-1 monocytic cell line was also activated by supernatants from activated T cells and CAR-T cells (Fig. 1D, E). To confirm that the IL-6 and IL-1β detected in myeloid and T cell co-culture supernatants originated from myeloid cells rather than from T cells or CAR-T cells, we assessed IL-6 and IL-1β levels in supernatants from unstimulated and CD3/CD28-activated T cells and CAR-T cells. IL-6 and IL-1β were not detectable in supernatants from either unstimulated or activated T cells or CAR-T cells (Fig. S1C). In contrast, IL-6 and IL-1β were detected only in supernatants from activated THP-1 cells and were not detected in unstimulated THP-1 supernatants (Fig. S1C). Collectively, these data indicate that the IL-6 and IL-1β measured in myeloid and T cell co-culture supernatants are derived from activated myeloid cells rather than from T cells or CAR-T cells.

Together, these data demonstrate that upon TCR-dependent, TCR-independent, or CAR-dependent activation, T and CAR-T cells release soluble factors that activate bystander myeloid cells.

### Comparative cytokine profiling of CAR-T cells following TCR- and CAR-dependent activation identifies inflammatory candidates

Since supernatants obtained from both TCR-dependent and CAR-dependent activation significantly induced myeloid cell activation (Fig. 1B–E), we hypothesized that shared inflammatory factors may play a central role in BMCA. To identify such candidates, we performed a comparative analysis of cytokines secreted by CAR-T cells following TCR- and CAR-mediated activation.

Anti-CD19 CAR-T cells were generated by activating peripheral blood-derived T cells with anti-CD3/CD28 antibodies, transducing them with an anti-CD19 CAR-expressing lentiviral vector, and expanding them ex vivo. The cell-free supernatant collected at harvest served as the TCR-dependent activation sample. For the CAR-dependent activation sample, anti-CD19 CAR-T cells were co-cultured with CD19⁺ Daudi cells at an effector-to-target (E: T) ratio of 1:1, and supernatant was collected after 24 h. Supernatants from unstimulated (US) CAR-T cells and Daudi cells were used as negative controls.

**Fig. 2:**
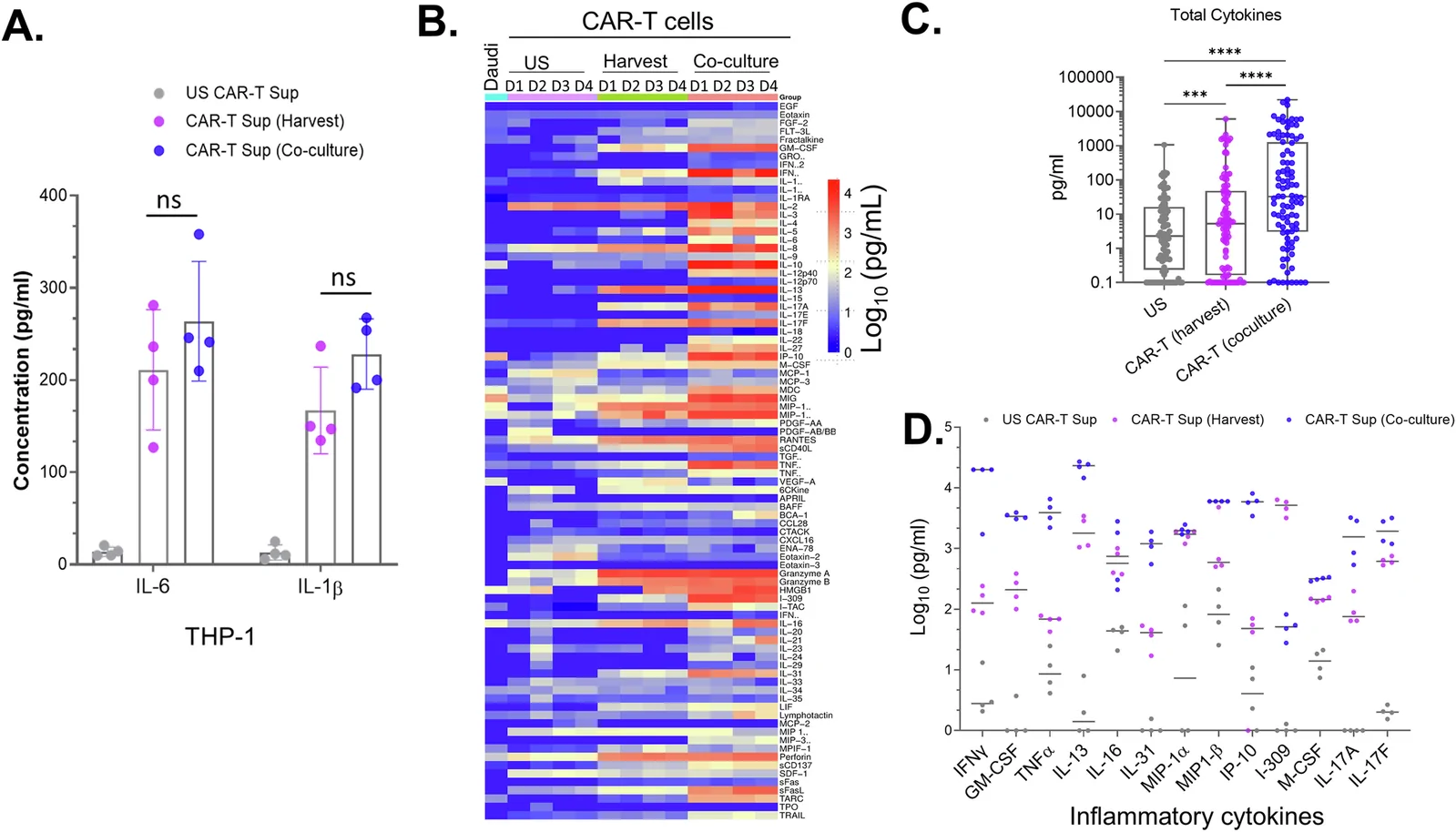
Comparative cytokine profiling of CAR-T cells following TCR- and CAR-dependent activation. **A** IL-6 and IL-1β secretion by the THP-1 monocytic cell line following treatment with cell-free supernatants obtained from either unstimulated (US) or activated anti-CD19 CAR-T cells. Supernatant collected during harvest of anti-CD19 CAR-T cells was used as the TCR-dependent activation sample, while supernatant collected following co-culture of anti-CD19 CAR-T cells with CD19⁺ Daudi cells was used as the CAR-dependent activation sample. **B** Heatmap of 96 inflammatory cytokines/chemokines present in supernatant samples obtained from CAR-T cells that were either unstimulated, TCR-activated with anti-CD3/CD28 (harvest), or CAR-activated with CD19⁺ Daudi cells (co-culture) at an effector-to-target ratio of 1:1. Cytokine/chemokine levels (expressed as log₁₀ pg/ml) are represented by a color gradient, ranging from blue (no expression) to red (highest concentration). Data from four donors (D1–D4) are shown. **C** Quantification of 96 cytokines/chemokines detected in the supernatant samples from panel **B**. Each dot represents a cytokine or chemokine, and the average concentration across four donors is shown. **D** Quantification of 13 inflammatory cytokines that were absent in the supernatant from US anti-CD19 CAR-T cells but present in the supernatant from TCR-activated (harvest) and CAR-activated (co-culture) anti-CD19 CAR-T cells. *P < 0.05, **P < 0.01, ***P < 0.001. ns= not significant. Each dot represents one donor.

Prior to cytokine profiling, the supernatants were tested for their ability to induce BMCA. As expected, only supernatants from activated CAR-T cells (both TCR-dependent and CAR-dependent), but not from US CAR-T cells, induced activation of THP-1 myeloid cells (Fig. 2A). We then subjected the supernatants to cytokine analysis. Supernatants from US CAR-T cells or Daudi cells contained minimal levels of inflammatory cytokines (Fig. 2B). In contrast, activation of CAR-T cells resulted in a significant increase in cytokine production, with consistent patterns observed across four independent donors (D1-D4) (Fig. 2B). Although both activation methods generated supernatants that robustly induced myeloid activation with no significant difference in potency (Fig. 2A), total inflammatory cytokine levels were significantly higher in the co-culture supernatant (CAR-dependent activation) than in the harvest supernatant (TCR-dependent activation) (Fig. 2C). We further analyzed 30 inflammatory cytokines individually and found that most were significantly upregulated following CAR-dependent activation compared with TCR-dependent activation (Fig. S2). Together, these data suggest that CAR-dependent activation may induce additional cytokines that do not directly contribute to BMCA.

Comparative analysis of cytokines present in the harvest and co-culture supernatants identified 13 cytokines, IFN-γ, GM-CSF, TNFα, IL-13, IL-16, IL-31, MIP-1α, MIP-1β, IP-10, I-309, M-CSF, IL-17A, and IL-17F, that were upregulated ( > 10-fold) relative to unstimulated controls and were common to both TCR- and CAR-dependent activation conditions (Fig. 2D). Because both activation methods induced myeloid cell activation, we focused on these 13 shared cytokines for further analysis, as they likely represent the primary contributors to inflammatory toxicities observed during CAR-T cell therapy.

In addition to these array-identified soluble cytokines, we next evaluated Glycoprotein Ibα (GPIbα) as an additional candidate mediator that was not included in the cytokine profiling panel but was implicated in myeloid activation in our prior CAR-NK studies [33], and we therefore tested whether it could similarly promote bystander myeloid activation following CAR-T cell activation.

### T cell-derived GPIbα activates myeloid cells

As stated above, since our cytokine profiling approach in Fig. 2 was limited to analytes included on the array, we next examined GPIbα, inflammatory mediator not represented in the cytokine array panel but implicated in myeloid cell activation in our prior CAR-NK studies [33], to assess its ability to induce bystander myeloid activation in the current CAR-T cell study.

(GPIbα) is a type I transmembrane protein and the major ligand-binding subunit of the GPIb-V-IX complex, best known for its role as a receptor for von Willebrand factor in hemostasis and thrombosis [34]. Recent studies have shown that soluble GPIbα secreted by activated lymphocytes (T and NK cells) can activate myeloid cells [33, 35].

**Fig. 3:**
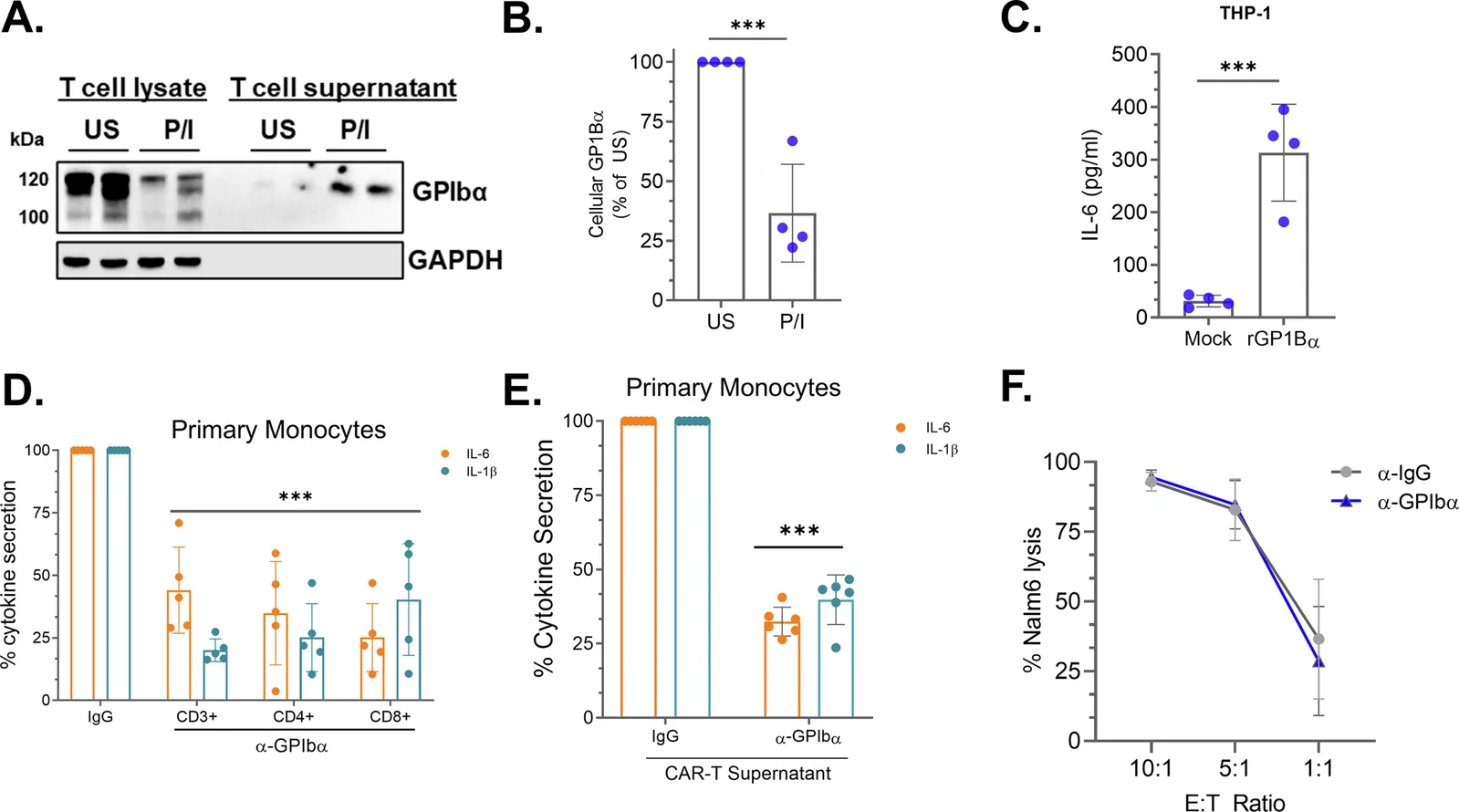
T cell-derived GPIbα activates myeloid cells. **A** Immunoblot analysis of cell lysates and supernatants obtained from unstimulated (US) or PMA/ionomycin-stimulated primary human T cells for GPIbα. GAPDH was used as a loading control. **B** Quantification of cellular GPIbα in lysates from US or PMA/ionomycin-stimulated primary human T cells. Percent (%) cellular GPIbα was normalized to US samples. **C** Recombinant rGPIbα protein was added to primary monocytes, and IL-6 release in the supernatant was measured by ELISA. Mock-treated primary monocytes received control rGFP protein. **D** Supernatants obtained from anti-CD3/CD28-activated primary human T cells (CD3⁺, CD4⁺, and CD8⁺) or **E** supernatants from Daudi co-culture–activated anti-CD19 CAR-T cells were subjected to neutralization using anti-GPIbα antibodies. Following neutralization, supernatants were added to primary monocytes from the same donor, and secretion of IL-6 and IL-1β by myeloid cells was measured. Percent (%) reduction in myeloid cell activation was calculated by normalizing IL-6 and IL-1β levels to those secreted by myeloid cells incubated with IgG control–treated supernatant. **F** Anti-CD19 CAR-T cell potency was assessed by measuring lysis of CD19⁺ Nalm6 target cells in the presence of control IgG or anti-GPIbα antibodies at various effector-to-target ratios. *P < 0.05, **P < 0.01, ***P < 0.001. Each dot represents one donor.

To assess whether human T cells express and secrete GPIbα, we analyzed both cell lysates and supernatants. Unstimulated (US) T cells expressed GPIbα intracellularly, as it was detected in cell lysates but not in supernatants (Fig. 3A), suggesting no active secretion under resting conditions. In contrast, upon activation, GPIbα was detected in the supernatant, and its intracellular levels were significantly reduced (Fig. 3A, B), indicating that T cell activation triggers GPIbα secretion.

To determine whether GPIbα directly contributes to myeloid cell activation, we treated primary human monocytes with recombinant GPIbα (rGPIbα). Consistent with prior findings [33], rGPIbα significantly induced IL-6 secretion from monocytes, confirming its inflammatory potential (Fig. 3C).

Next, we investigated whether neutralization of GPIbα secreted by activated T cells could suppress myeloid cell activation. CD3⁺, CD4⁺, and CD8⁺ T cells were isolated from peripheral blood and activated with anti-CD3/CD28 antibodies. Supernatants from these activated T cells were treated with either anti-GPIbα antibodies or control IgG before being applied to primary monocytes. GPIbα neutralization significantly reduced IL-6 and IL-1β secretion from monocytes across all T cell subsets compared with IgG-treated controls (Fig. 3D). These results suggest that GPIbα released by activated CD3⁺, CD4⁺, and CD8⁺ T cells contribute to bystander myeloid cell activation.

We then extended this analysis to CAR-T cells. Anti-CD19 CAR-T cells were activated by co-culture with CD19⁺ Daudi cells, and the resulting supernatants were treated with anti-GPIbα antibodies or control IgG. When applied to primary monocytes, GPIbα-neutralized supernatants led to a significant reduction in IL-6 and IL-1β production compared with IgG-treated controls (Fig. 3E), further confirming the inflammatory role of GPIbα.

Finally, to determine whether GPIbα neutralization impairs CAR-T cell function, we evaluated the cytotoxicity of anti-CD19 CAR-T cells against CD19⁺ Nalm6 cells in the presence of either control IgG or anti-GPIbα antibodies across a range of effector-to-target (E: T) ratios. GPIbα neutralization did not affect CAR-T cell cytotoxicity (Fig. 3F).

Together, these findings demonstrate that GPIbα is released by activated T cells and CAR-T cells, contributes to myeloid cell activation, and that its neutralization reduces inflammatory activation of myeloid cells without compromising CAR-T cell anti-tumor function in an in vitro short-term co-culture assay. Based on these results, we identify GPIbα as a key inflammatory mediator involved in bystander myeloid cell activation.

### Neutralization studies confirm the role of CAR-T cell secreted inflammatory factors in myeloid cell activation

A total of 14 soluble factors secreted by activated CAR-T cells were identified as potential contributors to myeloid cell activation (Figs. 2 and 3). To assess their functional relevance, we performed antibody-mediated neutralization of each factor in activated CAR-T cell supernatants. Anti-CD19 CAR-T cells from four independent donors were co-cultured with CD19⁺ Daudi cells to generate activated supernatants, and each sample was individually neutralized with factor-specific antibodies. Neutralized supernatants were incubated with myeloid cells for 24 h. IL-6 and IL-1β levels were measured as indicators of myeloid activation, with supernatants treated with non-specific IgG serving as controls. Antibody concentrations were optimized as previously described [33].

**Fig. 4:**
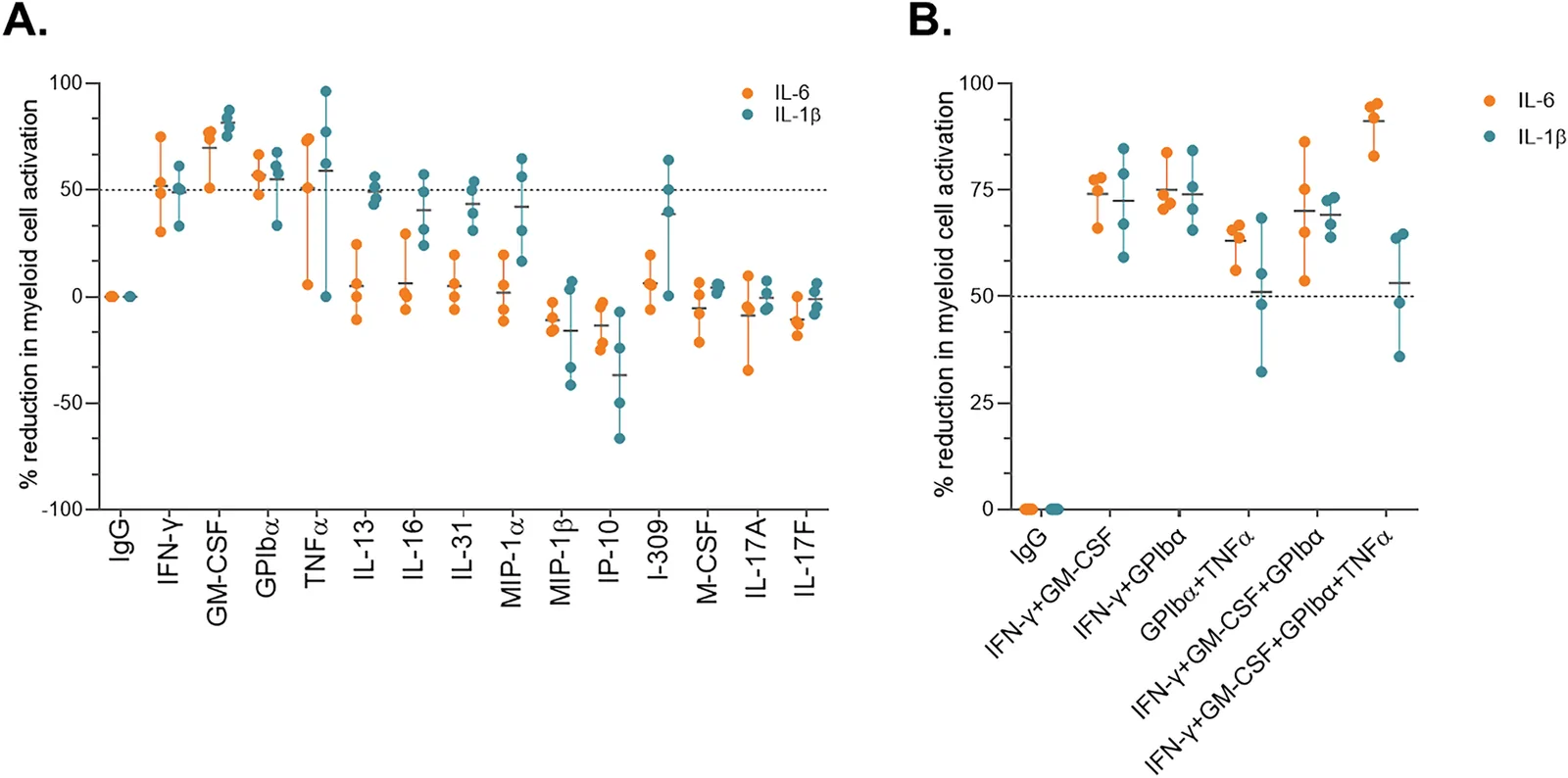
Neutralization studies identify the role of four CAR-T cell–secreted inflammatory factors in myeloid cell activation. Supernatants obtained from Daudi co-culture–activated anti-CD19 CAR-T cells (effector-to-target ratio of 1:1) were subjected to antibody-mediated neutralization. Following neutralization, the supernatants were added to THP-1 myeloid cells, and secretion of IL-6 and IL-1β was measured. Percent (%) reduction in myeloid cell activation was calculated by normalizing IL-6 and IL-1β levels to those secreted by myeloid cells incubated with IgG control–treated supernatants. Percent reduction in myeloid cell activation following neutralization of **A** 14 individual soluble inflammatory factors or **B** combinations of soluble inflammatory factors present in the supernatant from activated anti-CD19 CAR-T cells is shown. Each dot represents one donor.

Among the 14 factors, neutralization of IFNγ, GM-CSF, GPIbα, or TNFα each led to a ≥ 50% mean reduction in both IL-6 and IL-1β levels (Fig. 4A). In contrast, neutralization of IL-13, IL-16, IL-31, MIP-1α, and I-309 selectively reduced IL-1β but had minimal effect on IL-6. Neutralization of MIP-1β, IP-10, M-CSF, IL-17A, and IL-17F had little to no impact on either cytokine. These findings identify IFNγ, GM-CSF, GPIbα, and TNFα as major inflammatory mediators secreted by CAR-T cells that drive myeloid activation. Our results also confirm prior reports implicating IFNγ, GM-CSF and TNFα as key drivers of CAR-T-associated inflammation [21–26].

While each of the four major inflammatory factors, IFNγ, GM-CSF, GPIbα, and TNFα, contributed significantly to myeloid cell activation, neutralization of any single factor was insufficient to fully suppress this response (Fig. 4A). To evaluate potential synergistic effects, we performed combinatorial neutralization studies (Fig. 4B). Dual blockade of IFNγ and GM-CSF, or IFNγ and GPIbα, resulted in comparable reductions in myeloid activation (Fig. 4B), suggesting that GPIbα may contribute to inflammation at a level similar to GM-CSF. In contrast, combined neutralization of GPIbα and TNFα reduced IL-6 production to a similar degree but was notably less effective in suppressing IL-1β (Fig. 4B), indicating that IL-1β induction may rely more heavily on IFNγ and/or GM-CSF.

Addition of a third factor, IFNγ, GM-CSF, and GPIbα, did not further reduce cytokine levels compared to the dual combinations (Fig. 4B), implying that GM-CSF and GPIbα may function through overlapping or convergent pathways. However, simultaneous neutralization of all four factors (IFNγ, GM-CSF, GPIbα, and TNFα) led to a significantly greater reduction in IL-6 levels than any two- or three-factor combination (Fig. 4B), suggesting that comprehensive suppression of IL-6 requires broad inhibition of multiple inflammatory signals.

Paradoxically, this four-factor blockade was less effective in reducing IL-1β levels and mirrored the effect observed with dual neutralization of GPIbα and TNFα (Fig. 4B). These findings indicate that the regulation of IL-6 and IL-1β is governed by distinct cytokine networks, highlighting the complex and multifaceted nature of myeloid cell activation in response to CAR-T cell-derived factors.

Currently, CAR-T cell-associated inflammatory toxicities are primarily managed with corticosteroids or by inhibiting IL-6 and/or IL-1β signaling pathways using tocilizumab (an IL-6 receptor antagonist) or anakinra (an IL-1 receptor antagonist) [4]. In some cases, emapalumab (an anti-IFNγ antibody) or etanercept (a TNFα inhibitor), in combination with these agents, have also been used [19, 20]. Although these immunomodulatory therapies have shown efficacy in reducing CAR-T-related inflammatory toxicities, responses remain heterogeneous, and not all patients benefit [20, 36].

Consistent with this clinical variability, we observed donor-specific differences in the effects of neutralization on CAR-T cell supernatants. For example, in supernatants from donor 1, neutralization of IFN-γ and GM-CSF had minimal impact on IL-6 levels, whereas neutralization of GPIbα and TNFα reduced IL-6 by more than 60% (Fig. S3A). In donor 2, neutralization of IFN-γ and GM-CSF modestly reduced IL-6 (47%) but had minimal impact on IL-1β (8%), while neutralization of GPIbα and TNFα reduced IL-1β by 29% and IL-6 by 87% (Fig. S3B). In both donors, simultaneous neutralization of all four factors substantially reduced both IL-6 and IL-1β (Fig. S3).

Together, these data suggest that due to donor-specific variability, targeting individual inflammatory mediators may not be sufficient to suppress CAR-T-induced myeloid activation. A combinatorial strategy targeting multiple key mediators may therefore be necessary to achieve consistent and meaningful reductions in inflammatory toxicities across a broader patient population.

### siRNA-mediated knockdown of major inflammatory factors in CAR-T cells reduces myeloid cell activation

The identification of major inflammatory factors released by activated CAR-T cells that drive myeloid cell activation presents a novel opportunity to enhance safety by genetically modifying CAR-T cells during manufacturing. Although previous studies have identified roles for IFNγ, GM-CSF, and TNFα in mediating inflammatory toxicities during CAR-T cell therapy [22, 23, 26], none have evaluated the effect of simultaneous knockdown of these factors on myeloid cell activation. Furthermore, we demonstrated that GPIbα is a novel soluble factor released by activated CAR-T cells that significantly contributes to myeloid cell activation.

To assess whether knockdown of these four factors, IFN-γ, GM-CSF, TNFα, and GPIbα could reduce myeloid cell activation, anti-CD19 CAR-T cells were transfected with siRNAs targeting these mediators in three different combinations: IFN-γ + GM-CSF, GPIbα + TNFα, and all four factors combined. A non-specific siRNA served as a negative control. After 48 h, CAR-T cells were co-cultured with CD19⁺ Daudi cells. Following 24 h of co-culture, the resulting supernatants were incubated with primary monocytes, and myeloid cell activation was assessed by measuring IL-6 and IL-1β production.

**Fig. 5:**
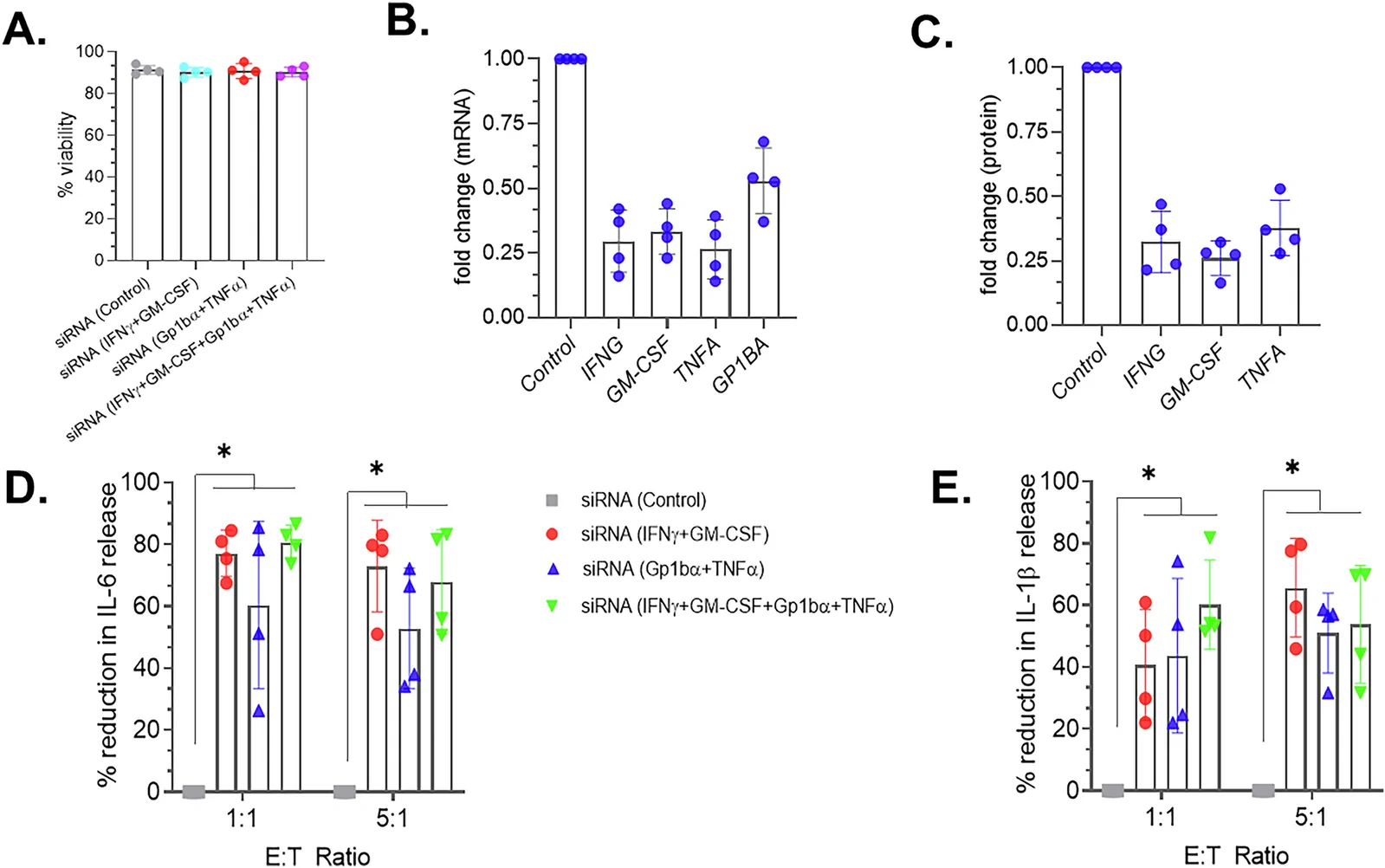
siRNA-mediated knockdown of four soluble factors in CAR-T cells reduces myeloid cell activation. Anti-CD19 CAR-T cells were transfected with the control non-specific siRNA or the pooled siRNAs to reduce GPIbα, GM-CSF, TNFα and IFNγ protein expression. **A** cellular viability, **B** mRNA and **C** protein expression was measured after 48 h of siRNA transfection. siRNA-treated anti-CD19 CAR-T cells were activated following co-culture with Daudi target cells at two different effector to target (E: T) ratios, 1:1 and 5:1. Myeloid (THP-1) cells were incubated with supernatants from siRNA-transfected and activated anti-CD19 CAR-T cells, and IL-6 and IL-1β secreted by myeloid cells were measured. Percent (%) reduction in myeloid cell activation was calculated by normalizing to the **D** IL-6 and **E** IL-1β levels secreted by myeloid cells incubated with control siRNA treated and activated anti-CD19 CAR-T cell supernatant. *P < 0.01. ns= not significant. Each dot represents one donor.

siRNA transfection had no impact on CAR-T cell viability (Fig. 5A). Across four donors, siRNA transfection significantly reduced mRNA expression, with average reductions ranging from 52% to 72% for the targeted genes (Fig. 5B). This was accompanied by significant decreases in protein expression of IFN-γ, GM-CSF, and TNFα, ranging from 63% to 74% (Fig. 5C). GPIbα protein levels were not quantified due to the lack of a suitable assay.

In all three combinations, knockdown of these inflammatory mediators in CAR-T cells significantly reduced IL-6 and IL-1β production by monocytes compared to control siRNA treated cells (Fig. 5D, E). No significant differences in cytokine reduction were observed among the three siRNA groups, suggesting that each combination is similarly effective at suppressing myeloid cell activation. While reductions were consistent across the four donors tested, some donor-to-donor variability was observed, as noted in the neutralization studies (Fig. S3). Thus, a combinatorial knockdown strategy targeting all four factors may offer the most broadly effective approach to mitigating CAR-T cell-mediated bystander myeloid cell activation.

**Fig. 6:**
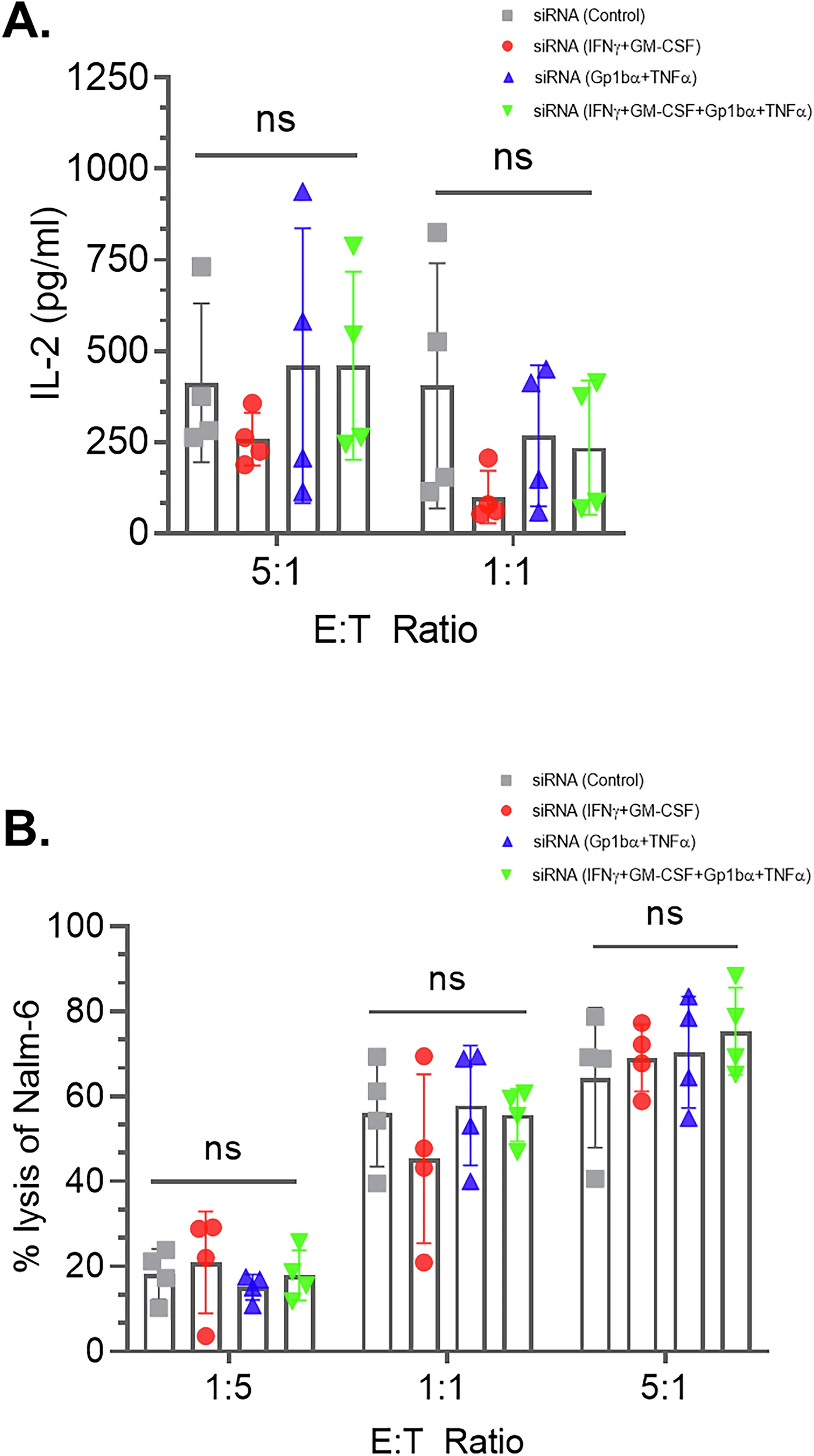
siRNA-mediated knockdown of four soluble factors in CAR-T cells does not affect CAR-T cell potency. Potency of siRNA-transfected anti-CD19 CAR-T cells was assessed by cytokine secretion and cytotoxicity. **A** Following co-culture of siRNA-transfected anti-CD19 CAR-T cells with CD19⁺ Daudi cells at effector-to-target (E: T) ratios of 1:1 and 5:1 for 24 h, IL-2 secretion by CAR-T cells was quantified by ELISA. **B** Cytotoxicity of siRNA-transfected anti-CD19 CAR-T cells was assessed by measuring lysis of CD19⁺ Nalm6 target cells after 24 h of co-culture at low (1:5), medium (1:1) and high (5:1) E: T ratios. ns = not significant. Each dot represents an individual donor.

It is important to note that the magnitude of the effects observed in our in vitro studies will need to be confirmed in in vivo and other relevant models. Incomplete reduction in myeloid cell activation suggests that these mediators contribute to bystander myeloid activation but are unlikely to function in isolation, consistent with redundancy and compensatory signaling within inflammatory networks. While these data support a contributory role for IFN-γ, GM-CSF, GPIbα, and TNF-α in CAR T-mediated myeloid activation, the modest effect sizes indicate that additional studies are required to achieve more robust mitigation of toxicity. Importantly, our in vitro assays model early, short-term interactions and may underestimate or fail to capture amplifying effects that could occur in vivo or after repeated antigen exposure. Therefore, the translational impact of targeting these factors will require validation in more physiologic models that assess durability, persistence, and systemic inflammatory responses. To evaluate whether gene silencing of inflammatory mediators affects CAR-T cell function, we assessed two key indicators of CAR-T potency: cytokine secretion and cytotoxic activity. Anti-CD19 CAR-T cells transfected with siRNAs targeting IFN-γ, GM-CSF, TNFα, and GPIbα (in the three combinations) were co-cultured with CD19⁺ Daudi cells at two different effector-to-target (E: T) ratios. IL-2 secretion, a marker of CAR-T cell activation and function, was measured 24 h post co-culture. We selected IL-2 instead of IFNγ for potency assessment because measurement of IFNγ in this setting would primarily reflect siRNA knockdown efficiency rather than CAR-T cell potency, since the cells were treated with a pooled siRNA that included IFNγ-specific siRNA. No significant differences in IL-2 levels were observed between gene-specific siRNA-transfected CAR-T cells and those transfected with control siRNA (Fig. 6A), indicating preserved CAR-T cell activation.

To further assess cytotoxic function, we evaluated the ability of siRNA-transfected CAR-T cells to lyse CD19⁺ Nalm6 target cells at low (1:5), medium (1:1) and high (5:1) E:T ratios. Including a low E:T ratio provides a more stringent assessment of CAR-T cytotoxic function because limited effector numbers can reveal subtle impairments that may be masked at higher ratios. No significant differences in cytotoxic activity were observed between gene-silenced and control CAR-T cells across multiple donors and various E:T ratios (Fig. 6B). Notably, we observed comparable target-cell killing at an E:T ratio of 1:5 following combined downregulation of IFN-γ, GM-CSF, GPIbα, and TNF-α, supporting the conclusion that suppressing these inflammatory mediators does not measurably compromise short-term CAR-T cytotoxic potency under in vitro conditions.

To assess whether siRNA delivery and cytokine gene silencing affect CAR-T cell fitness beyond short-term cytotoxicity, we evaluated CAR-T viability and proliferative capacity following antigen engagement. siRNA-transfected CAR-T cells were stimulated with CD19⁺ target cells and monitored over multiple time points for viability and expansion, in comparison with control siRNA-treated CAR-T cells. Under our experimental conditions, siRNA delivery and cytokine gene silencing did not significantly alter CAR-T viability, proliferation, expansion kinetics, or overall cell yield (Fig. S4).

Together, these findings demonstrate that simultaneous siRNA-mediated knockdown of key inflammatory mediators, IFN-γ, GM-CSF, TNFα, and GPIbα does not impair CAR-T cell potency in an in vitro short-term co-culture assay. These results support the feasibility of using gene silencing strategies to mitigate inflammatory toxicities without compromising the therapeutic efficacy of CAR-T cells.

## Discussion

CAR-T cell therapies are associated with life-threatening inflammatory toxicities, including cytokine release syndrome (CRS) and immune effector cell-associated neurotoxicity syndrome (ICANS) [1, 37, 38]. The mechanisms underlying these toxicities are complex and likely involves multiple factors [4]. Upon binding to CAR-specific antigens expressed by target cells, CAR-T cells become activated, proliferate, and release soluble factors [2, 4]. These soluble mediators can activate bystander myeloid cells, which in turn secrete pro-inflammatory cytokines such as IL-6 and IL-1β, central drivers of CAR-T-associated inflammatory toxicities [15, 20, 22, 23, 25, 26, 39]. Consequently, therapies such as tocilizumab (an IL-6 receptor antagonist) and anakinra (an IL-1 receptor antagonist) have been employed to manage these toxicities in patients [4, 15]. We did not assess other inflammatory mediators secreted by activated myeloid cells, such as prostaglandin E2, in this study, as their direct contribution to CAR-T associated inflammatory toxicities remain incompletely defined.

Our study demonstrates that CAR-T-derived cytokines are particularly potent in promoting excessive myeloid cell activation. Using comprehensive cytokine profiling, we identified 14 soluble factors involved in bystander myeloid cell activation (BMCA), with IFN-γ, GM-CSF, TNFα, and GPIbα playing dominant roles. Supernatants from activated T cells and CAR-T cells robustly induced IL-6 and IL-1β secretion from myeloid cells, confirming their pro-inflammatory potential. Unstimulated T cells did not induce BMCA, underscoring the requirement for activation in initiating this response.

A recent study reported that soluble factors released by CAR-NK cells also trigger BMCA, though at significantly lower levels than CAR-T cells [33]. That study identified soluble GPIbα, GM-CSF, TNFα, and MIP-1α as the major drivers of CAR-NK–mediated BMCA. In our study, GPIbα, GM-CSF, TNFα, and IFN-γ emerged as the dominant CAR-T-derived inflammatory factors. Thus, three of the four factors, GPIbα, GM-CSF, and TNFα, are shared between CAR-T and CAR-NK cells, suggesting they represent common lymphocyte-derived mediators of BMCA. Interestingly, neutralization studies revealed differences: in CAR-NK cells, blocking IFN-γ had minimal impact on IL-6 and IL-1β secretion [33], while in CAR-T cells, blocking MIP-1α had no effect on IL-6 secretion and only partially reduced IL-1β secretion (Fig. 4A). Together, these findings suggest that while certain inflammatory factors are shared drivers of BMCA across lymphocyte lineages, others play lineage-specific roles in mediating this response.

To mitigate BMCA-driven inflammation, we explored single and combinatorial cytokine neutralization strategies. Neutralization of individual factors, IFN-γ, GM-CSF, TNFα, and GPIbα reduced IL-6 and IL-1β production by myeloid cells, although considerable donor-to-donor variability was observed. In donors where neutralization of IFN-γ and GM-CSF had minimal effect, targeting TNFα and GPIbα produced a significant reduction. Conversely, in some donors, neutralization of IFN-γ and GM-CSF was comparable to TNFα and GPIbα in reducing IL-6, suggesting that when IFN-γ cannot be effectively targeted, alternative strategies focusing on TNFα and GPIbα may be viable. Collectively, these data indicate that targeting individual factors may not be sufficient to broadly mitigate CAR-T-induced inflammatory toxicities across a diverse patient population, and targeting all four factors may enhance CAR-T safety. Importantly, simultaneous neutralization all four factors did not impair CAR-T cell tumor-killing efficacy, supporting this approach as a viable strategy to reduce therapy-associated toxicities.

siRNA-based approaches to enhance CAR-T cell function have been extensively studied, such as targeting TGF-β to overcome immunosuppressive tumor microenvironments or silencing HLA genes to reduce graft-versus-host disease risk in allogeneic CAR-T cells [40, 41]. In our study, siRNA-mediated knockdown of IFN-γ, GM-CSF, TNFα, and GPIbα confirmed their role in driving myeloid cell activation, highlighting the potential of multi-targeting approaches over single-factor interventions. Importantly, knockdown of these factors did not affect CAR-T cell viability, IL-2 production, or cytotoxicity. These findings also extend prior studies demonstrating the utility of siRNA in enhancing CAR-T therapy by targeting immunosuppressive and inflammatory pathways [40, 41].

Our findings should be interpreted in the context of several translational considerations and limitations. First, the relative contribution of IFN-γ, GM-CSF, TNFα, and GPIbα to inflammatory toxicity may be context dependent and vary across tumor types, antigen targets, and microenvironments. Notably, IFN-γ signaling has been reported to enhance CAR-T activity in solid tumor settings by increasing tumor-CAR-T adhesion and synapse stability, whereas it appears less critical in leukemia/lymphoma models [27]. Because our cytotoxicity studies were performed using the leukemia-derived Nalm6 cell line, these in vitro findings should not be broadly generalized to other tumor contexts, and additional studies will be needed to evaluate the impact of IFN-γ modulation across diverse disease settings. Second, inflammatory signaling is highly redundant, and the modest reductions observed with single-factor blockade are consistent with compensatory pathways that may sustain myeloid cell activation. Finally, our experiments were conducted primarily in short-term in vitro co-culture systems and did not assess repeated antigen stimulation, long-term persistence, or in vivo efficacy/toxicity. Future studies incorporating repeated stimulation paradigms and relevant in vivo models will be required to determine whether combinatorial targeting of these mediators can provide durable mitigation of CRS/ICANS while preserving CAR-T expansion, persistence, and function. It is also important to note that considerable inter-donor variability was observed following antibody-mediated neutralization or siRNA-mediated knock down of inflammatory factors in reducing myeloid cell activation. Although, this may, in part, be due to incomplete neutralization or knock down of inflammatory factors, underlying genetic differences in cytokine receptor signaling pathways may also contribute to this effect. For example, polymorphisms have been reported in the IFN-γ receptor genes IFNGR1 and IFNGR2, as well as in the GM-CSF receptor genes CSF2RA and CSF2RB, within the general population [42–45]. Although common variants in these receptors have not been definitively linked to altered sensitivity to IFN-γ or GM-CSF in the context of CAR-T-associated inflammation, genetic differences influencing receptor expression levels, ligand binding affinity, or downstream signaling efficiency could theoretically modulate the magnitude of myeloid activation. Future studies incorporating genotypic analysis may help clarify the contribution of receptor polymorphisms to variability in inflammatory responses.

In conclusion, our study identifies IFN-γ, GM-CSF, TNFα, and GPIbα as key inflammatory mediators released by activated CAR-T cells that drive bystander myeloid cell activation and contribute to therapy-associated toxicities such as CRS and ICANS. Both neutralization and siRNA-mediated knockdown of these factors effectively reduced myeloid activation without compromising CAR-T cell viability, cytokine production, or cytotoxic function, highlighting the potential of multi-targeting strategies to improve the safety profile of CAR-T therapies. Importantly, donor-to-donor variability in cytokine responses underscores the need for personalized approaches in CAR-T cell design and manufacturing. Collectively, these findings provide a mechanistic framework for mitigating CAR-T-induced inflammatory toxicities and support the development of combinatorial or gene-silencing interventions to enhance the safety and efficacy of next-generation CAR-T therapies.

## Supplementary information

Supplemental Figure S1

Supplemental Figure S2

Supplemental Figure S4

Supplemental Information

Supplemental Figure S3

## Data Availability

All data generated in this study are included in the published article and the supplement section of the manuscript.
