# Supplementary material for: Characterization of CAR-T cell factors that contribute to myeloid cell activation
Source: Gene Ther. 2026 May 26;33(5):616–27. doi: 10.1038/s41434-026-00620-6 (PMC13585559; doi:10.1038/s41434-026-00620-6)
Supplement: Supplementary file 1 [file 41434_2026_620_MOESM4_ESM.docx]

**Supplementary information**

**Supplementary Figure S1. Activation of human T cells and CAR-T cells.**

**(A)** Primary human T cells were activated either with anti-CD3/CD28 antibodies (TCR-dependent) or with PMA/ionomycin (TCR-independent). **(B)** Anti-CD19 CAR-T cells were activated by co-culture with CD19⁺ Daudi cells. IFN-γ and GM-CSF levels in culture supernatants were measured by ELISA after 24 hours. **(C)** IL-6 and IL-1β levels were measured in supernatants from unstimulated (US) CAR-T cells, CD3/CD28-activated CAR-T cells, unstimulated THP-1 cells, and LPS-stimulated THP-1 cells (positive control). Each dot represents an individual donor.

**Supplementary Figure S2. Differential cytokine expression following TCR- and CAR-dependent activation.**

Inflammatory cytokine and chemokine levels were measured in supernatants from CAR-T cells activated either through the TCR using anti-CD3/CD28 stimulation (collected at harvest) or through CAR engagement via co-culture with CD19⁺ Daudi cells for 24 hours at an effector-to-target (E: T) ratio of 1:1. Data from four independent donors are shown for each cytokine and chemokine. Each dot represents an individual donor, and connecting lines indicate paired measurements from the same donor under the two activation conditions.

**Supplementary Figure S3. Donor-to-donor variability in neutralization of inflammatory factors in supernatants from activated CAR-T cells.**

Anti-CD19 CAR-T cells from two donors (Donor 1 and Donor 2) were activated by co-culture with CD19⁺ Daudi cells at an effector-to-target (E: T) ratio of 1:1. After 24 hours, cell-free supernatants were collected and subjected to antibody-mediated neutralization. Three antibody combinations were tested: IFN-γ + GM-CSF, GPIbα + TNFα, and IFN-γ + GM-CSF + GPIbα + TNFα. Non-specific IgG served as a negative control. Neutralized supernatants were then incubated with THP-1 cells for 24 hours, and **(A)** IL-6 and **(B)** IL-1β levels produced by THP-1 cells were quantified by ELISA.

**Supplementary Figure S4. siRNA-mediated knockdown of soluble factors does not affect CAR-T cell viability or proliferation.**

The impact of siRNA transfection on CAR-T cells was evaluated by measuring viability and proliferation following siRNA treatment. Three siRNA combinations were tested: IFN-γ + GM-CSF, GPIbα + TNFα, and IFN-γ + GM-CSF + GPIbα + TNFα. Non-specific control siRNA served as a negative control. **(A)** Cell viability was assessed using an automated cell counter at Day 0 (pre-transfection), Day 1 (24 hours post-transfection), Day 4 (72 hours post-transfection), and Day 6 (5 days post-transfection). **(B)** Proliferation was measured by counting viable cells following siRNA treatment using an automated cell counter. ns, not significant. Each dot represents an individual donor.
